# Morphological and functional aspects of progenitors perturbed in cortical malformations

**DOI:** 10.3389/fncel.2015.00030

**Published:** 2015-02-12

**Authors:** Sara Bizzotto, Fiona Francis

**Affiliations:** ^1^INSERM UMRS 839Paris, France; ^2^Sorbonne Universités, Université Pierre et Marie CurieParis, France; ^3^Institut du Fer à MoulinParis, France

**Keywords:** neurodevelopment, mouse mutant, radial glial cells, proliferation, epilepsy, intellectual disability, lamination

## Abstract

In this review, we discuss molecular and cellular mechanisms important for the function of neuronal progenitors during development, revealed by their perturbation in different cortical malformations. We focus on a class of neuronal progenitors, radial glial cells (RGCs), which are renowned for their unique morphological and behavioral characteristics, constituting a key element during the development of the mammalian cerebral cortex. We describe how the particular morphology of these cells is related to their roles in the orchestration of cortical development and their influence on other progenitor types and post-mitotic neurons. Important for disease mechanisms, we overview what is currently known about RGC cellular components, cytoskeletal mechanisms, signaling pathways and cell cycle characteristics, focusing on how defects lead to abnormal development and cortical malformation phenotypes. The multiple recent entry points from human genetics and animal models are contributing to our understanding of this important cell type. Combining data from phenotypes in the mouse reveals molecules which potentially act in common pathways. Going beyond this, we discuss future directions that may provide new data in this expanding area.

## Introduction

**Figure 1:**
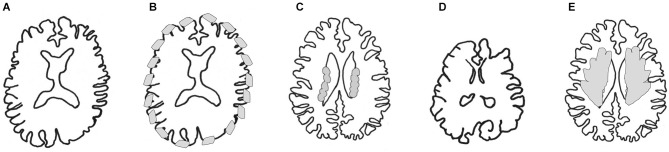
**MRI schemas of malformations. (A)** Control brain, **(B)** Cobblestone lissencephaly, where neuronal overmigration (represented by gray patches at the surface of the brain) can arise due to breaks of the basement membrane. **(C)** Periventricular nodular heterotopia, some neurons (represented by gray nodules) remain stuck at the ventricular surface, most probably due to breaks and disorganization of the ventricular lining. **(D)** Microcephaly, several mechanisms may give rise to this malformation leading to a greatly reduced size of the brain. In pure forms, brain architecture is relatively well-preserved, in other forms (microcephaly with simplified gyral pattern, MSGP, not shown), brain organization and cortical folds are also affected. **(E)** Globular or ribbon-like heterotopia, represented by gray globular masses. In this case the heterotopia starts at the level of the ventricles and fills up the white matter in some brain areas. The heterotopia can appear to have gyri. Modified from Francis et al. (2006).

Cortical malformations (Figure 1) are usually detected during pregnancy (fetal ultrasound), and are obvious after birth due to developmental delay, epilepsy and intellectual deficits. In human, magnetic resonance imaging (MRI) is used to classify the defects and if a genetic origin is suspected, this classification directs potential genetic screens. New variants of these disorders, unexplained by known genes, are currently the subject of exome sequencing projects. Studies in the mouse, as well as in other organisms, try to model these disorders. Knockdown or knockout of genes of interest reveals the cellular mechanisms. Alternatively, mouse mutants arise spontaneously and their characterization subsequently helps reveal both new genes and mechanisms. In general there are many different forms of cortical malformation, and many variants in each category. This review aims not to be exhaustive, but to resume general notions related to the abnormal functioning of progenitor cells. We start here by briefly describing the malformations of interest at the morphological level. We then group different gene mutations, classifying by similar phenotypes observed in mouse mutants, and in so-doing, dissect different aspects of progenitor cell function. Finally, we discuss and integrate all this information in order to have a more global current view of the cellular mechanisms related to malformations.

## Cortical malformations

For so-called disorders of neuronal migration, neurons derived from zones of proliferation close to the ventricles, do not reach their correct destination in the cortical plate (CP), either because they are arrested in the white matter (subcortical band heterotopia, SBH or “double-cortex”, online mendelian inheritance in man OMIM 300067), and/or form a disordered, often thickened, CP (Barkovich et al., 2012). A thicker cortex is often associated with abnormal cortical gyri, leading either to a simplified or abnormal gyral pattern or, in their absence, to a smooth appearance of the cortical surface (lissencephaly). The type 1 lissencephaly spectrum (e.g., OMIM 607432, 300067) hence includes a smooth, thickened and disorganized cortex (agyria), or simplified, thickened and abnormal gyri (pachygyria), or SBH. X-linked inheritance, different gene mutations and different genes (Table 1; Des Portes et al., 1998; Jaglin and Chelly, 2009) explain the spectrum of phenotypes. In general these latter disorders may involve intrinsic functions in migrating neurons which are not mentioned here, although some genes play multiple roles including in neural progenitors, which we discuss in Section Mouse and Human Mutations and Mechanisms Important for RGC Function.

**Table 1:** **Genes and human malformations**.

Malformation
Microcephaly^1^	ASPM^2^	CDK5RAP2	MCPH1	CENPJ	WDR62^2^	STIL	CEP152	CEP63
MSGP^1^	WDR62^2^	NDE1	TUBB3^2^	ASPM^2^	KIF5C^2^
Syndrome including microcephaly (e.g., complex MCD^3^ or associated with PMG^4^)	TBR2	CENPE	DYNC1H1	TUBG1	KIF2A	KIF5C^2^	PLK4
Periventricular heterotopia	FLNA	ARFGEF2	C6orf70	FAT4	DCHS1
Lissencephaly (type I)	LIS1	DCX	TUBA1A	TUBB3^2^	RELN
PMG^4^	TUBB2B	GPR56	TUBA8	TUBB3^2^	TUBA1^2^A	WDR62^2^	NHEJ1	KBP
Lissencephaly (type II)	FKTN	POMT1	POMT2	POMGNT1	FKRP	LARGE	LAMB1
Atypical heterotopia^5^	EML1

*^1^All genes code for proteins with centrosomal-related activities, MSGP, microcephaly with simplified gyral pattern; ^2^Some genes appear in multiple categories depending on the patient and gene mutation; ^3^Complex malformation of cortical development (MCD) associated with microcephaly; ^4^PMG, Polymicrogyria; ^5^Associated with macrocephaly and sometimes hydrocephaly*.

Overmigration of neurons at the pial surface (so-called cobblestone or type II lissencephaly, e.g., OMIM 236670, 253800, Cormand et al., 2001; van Reeuwijk et al., 2005), represents a different set of disorders involving perturbed progenitors (Section Basal Processes of RGCs, Shedding Light on Heterotopias, Polymicrogyria and Type II Lissencephaly). Walker Warburg syndrome (WWS) is a severe autosomal recessive disorder of this nature characterized by muscular dystrophy, eye and neuronal migration defects. Overmigration gives rise to disorganized cerebral and cerebellar cortices and multiple coarse gyri with agyric regions (cobblestone lissencephaly). As well as this, the structural brain anomalies include agenesis of the corpus callosum, cerebellar hypoplasia and hydrocephalus. WWS is grouped within a series of disorders which include Fukuyama congenital muscular dystrophy (FCMD; Kobayashi et al., 1998), and Muscle-Eye-Brain disease (MEB). Mutations in a number of related genes have been associated with the various types of cobblestone lissencephaly (Section Basal Processes of RGCs, Shedding Light on Heterotopias, Polymicrogyria and Type II Lissencephaly, Table 1; Godfrey et al., 2007).

Polymicrogyria (e.g., OMIM 615752, 610031, 606854, Leventer et al., 2010) is considered as a separate entity, although there can be overlapping features with cobblestone lissencephaly (Bahi-Buisson et al., 2010). This disorder is characterized by multiple small folds at the surface of the brain, either diffuse or restricted to one brain region. The mechanisms causing this disorder initially remained elusive and they have for some time been described as affecting the end stages of migration. With the identification of various mutant genes (Table 1; Squier and Jansen, 2014), and studies in the rodent, it has become clear that a number of genes play a role in progenitors (Section Basal Processes of RGCs, Shedding Light on Heterotopias, Polymicrogyria and Type II Lissencephaly), especially the end, basal attachment of radial glial cells (RGCs) forming the pial surface of the cortex.

Periventricular heterotopia (PH, e.g., OMIM 300049, Parrini et al., 2006) is associated with large clusters of neurons present at the ventricular surface. These are often observed on MRI as visible gray matter nodules extending into the ventricles. Since neurons are known to be generated in these regions during development, it is assumed that some neurons, after being produced, do not migrate at all. Mouse models reveal abnormalities at the ventricular lining, which is made up of apical RGC end-feet with intricate cell-cell junctions (Section Apical Adhesive Interactions and Mechano-Transduction, Shedding Light on PH and Ciliopathies). Each of the PH genes (Table 1; Sheen, 2014), shows potential roles linking the plasma membrane to the cytoskeleton and some of these genes may also be important during neuronal migration.

Hydrocephaly (e.g., OMIM 307000) is associated with an abnormal quantity of cerebrospinal fluid in the ventricles, causing them to be larger than normal. Genetic causes related to specific human hydrocephaly phenotypes are still relatively unknown, with the notable exception of L1-CAM mutations (Adle-Biassette et al., 2013), involved in a syndrome including hydrocephalus due to aqueductal stenosis. Although causative mechanisms are indeed heterogeneous, hydrocephaly can arise because fluid movement is impaired. One defect is related to motile cilia on neuroependymal cells, as ciliary beating drives fluid flow (Tissir et al., 2010; Tong et al., 2014). Mouse mutations that affect motile ciliogenesis can thus lead to hydrocephalus, disruptions in neurogenesis and brain tumor formation (Han et al., 2009; Tissir et al., 2010; Hildebrandt et al., 2011). Primary non-motile cilia are known to act as mechano-transducers, transmitting signals to the developing tissue (Paridaen et al., 2013). Abnormalities in these processes may or may not be associated with hydrocephaly. Abnormal cilia, and the cycle where cilia components are disassembled to be re-used during mitosis, can also be associated with other cortical malformations. Various mouse mutants with progenitor defects show hydrocephaly (Sections Apical Adhesive Interactions and Mechano-Transduction, Shedding Light on PH and Ciliopathies, Table 2), although often the exact causes of this remain unidentified.

**Table 2:** **Specific genes mouse mutants**.

Gene (Protein)	Animal model	Main RGC phenotype	Main cortical phenotype	Corresponding human malformation	Reference
*Gpr56* (Gpr56)	Knockout mouse	Disruption of basal attachment	Irregular thickness and organization; Reduced number of VZ and SVZ progenitors at E14.5	Restricted polymicrogyria; Cobblestone lissencephaly-like neuronal over-migration	Bae et al. (2014)
	Transgenic overexpression of human *GPR56* in mouse		Increased VZ and SVZ progenitors		Bae et al. (2014)
*Itgb1* (β1-integrin)	*Nestin-Cre* conditional knockout mouse	Lack of basal glial endfeet; irregular glial fibers	Disorganized cortical layers at E15.5; Ectopic neurons in the MZ and deep in the cortical wall; Defective meningeal basement membrane		Graus-Porta et al. (2001)
	Antibody-mediated blocking at E12.5 and E15.5 in mouse cortex	Progenitors divide outside the VZ; Apical process detachment; Dystrophic basal processes	Reduction in the width of cortical layers I-V		Loulier et al. (2009)
*Itga* (α-integrin)	α6-integrin knockout mouse	Lack of basal glial endfeet; irregular glial fibers	Disorganized cortical layers from E13.5 to E18.5; Ectopic neurons in the MZ and deep in the cortical wall; Defective meningeal basement membrane		Georges-Labouesse et al. (1998)
	α3- and α6-integrin double knockout mouse		Disorganized cortical layers from E13.5 to E16.5; Ectopic neuroblastic outgrowths		De Arcangelis et al. (1999)
*Fak* (Fak)	*Emx1-Cre* conditional knockout mouse	Defective basal glial endfeet; irregular glial fibers	Neuronal ectopia in and above the MZ from E14.5; Disorganized cortical layers; Disrupted basement membrane		Beggs et al. (2003)
*Ilk* (Ilk)	*Emx1-Cre* conditional knockout mouse	Defective basal glial endfeet; disorganized glial fibers	Cortical lamination defects from E14.5; Neuronal ectopia in the MZ; Basal lamina fragmentation; Defective positioning of Cajal-Retzius cells		Niewmierzycka et al. (2005)
*Rhoa* (RhoA)	*Emx1-Cre* conditional knockout mouse	Disorganized basal processes; neuronal ectopia in MZ; Loss of apical anchoring; Mitotic cells scattered ectopically	Subcortical band heterotopia (SBH); Cobblestone lissencephaly-like neuronal over-migration;		Cappello et al. (2012)
*Marcks* (Marcks)	Knockout mouse	Disrupted radial glial scaffold at E15.5; Abnormal radial glial endfeet; Disrupted apical polarity components; Disrupted mitotic orientation;	No clear delineation of cortical layers; Cobblestone lissencephaly; Disrupted VZ; Ectopic progenitors; Reduced cellular density and thickness of both VZ and SVZ		Weimer et al. (2009)
*Lama2* (Laminin-α2)	Knockout mouse	Progenitors dividing outside the VZ; Apical process detachment; Dystrophic basal processes			Miyagoe et al. (1997); Loulier et al. (2009)
*Mltt4 or Cdh2 (Afadin and Cdh2)*	*Emx1-Cre* conditional knockout mouse	Disruption in adherens junctions, Progenitors divide outside the VZ; Shorter cell cycle and reduced cell cycle exit	Double cortex-like phenotype		Gil-Sanz et al. (2014)
*FlnA* (FlnA)	Knockout mouse	Loss of adherens junctions	Focal disruptions of the VZ/SVZ and cell expansion into the ventricular space; Disruptions of the VZ surface	Periventricular heterotopia (PH)	Feng et al. (2006)
*Mekk4* (Mekk4)	Knockout mouse		Periventricular heterotopia (PH); Focal disruptions of the VZ/SVZ and cell expansion into the ventricular space; Thinner IZ; Subpial ectopia and polymicrogyria; Decrease in CP thickness;		Sarkisian et al. (2006)
	E14.5 mouse RNAi-mediated knockdown by *in utero* electroporation	Laminin disruption-mediated disorganization of radial glial fibers			Sarkisian et al. (2006)
*Fat4* (Fat4)	E13.5 and E14.5 mouse RNAi-mediated knockdown by *in utero* electroporation	Increased progenitor proliferation in VZ and SVZ; Block in differentiation between the Pax6+ and Tbr2+ states	Progenitor displacement, accumulation of neural precursors, and periventricular heterotopia (PH)	Van Maldergem syndrome with a partially penetrant PH phenotype	Cappello et al. (2013)
*Dchs1* (Dchs1)	E13.5 and E14.5 mouse RNAi-mediated knockdown by *in utero* electroporation	Increased progenitor proliferation in VZ and SVZ; Block in differentiation between the Pax6+ and Tbr2+ states	Progenitor displacement, accumulation of neural precursors, and periventricular heterotopia (PH)	Van Maldergem syndrome with a partially penetrant PH phenotype	Cappello et al. (2013)
*Efnb1* (Ephrin-B1)	E13.5 mouse overexpression by *in utero* electroporation	Blocking in progenitor differentiation			Qiu et al. (2008)
	E13.5 mouse RNAi-mediated knockdown by *in utero* electroporation	Increased progenitor differentiation			Qiu et al. (2008)
	Knockout mouse	Loss of RGC radial organization; Reduced progenitor number	Irregular appearance with micro-invaginations at the apical surface of the neuroepithelium; Misplacement of mitotic nuclei within the cortical wall		Davy et al. (2004); Qiu et al. (2008); Arvanitis et al. (2013)
*Ar13b* (Arl13b)	Knockout mouse (null allele)	RGC inverted polarity; RGC somas ectopically located near the pial surface; ciliary-based signaling perturbed	Neurons ectopically located near the ventricular surface; Reversed Reelin localization; Discontinuous pial membrane; Disrupted apical adherens junctions; Marked disruption of neuronal layer organization	Joubert syndrome with disrupted neurogenesis and malformed cerebral cortex	Cantagrel et al. (2008); Higginbotham et al. (2013)
	*FoxG1-Cre* conditional knockout mouse	Abnormal organization of the RGC scaffold; Loss of RGC apical-basal polarity	Perturbed neuronal positioning and layer formation		Higginbotham et al. (2013)
α-E-catenin (α-E-catenin)	*Emx1-Cre* conditional knockout mouse	Disruption of radial glial morphology and ventricular lining architecture; Formation of rosette-like structures	Formation of a large SBH and thinner layered cortex		Schmid et al. (2014)
	*Nestin-Cre* conditional knockout mouse	Disruption of RGC apical-junctional complexes; Loss of RGC polarity; At E13.5 abnormal activation of the Shh pathway causing: cell cycle shortening, increased number of mitotic cells, decreased apoptosis	Massive dysplasia; VZ cell dispersion; Increase in cortical thickness and size		Lien et al. (2006)
*Ctnnb1* (β-catenin)	*FoxG1-Cre* conditional knockout mouse	Disruption of progenitor apical adherens junctions	Telencephalon size severely reduced at E10.5; Loss of neuroepithelial integrity; Progenitor delamination and apoptosis		Junghans et al. (2005)
	Transgenic overexpression in mouse progenitors		Tangential increase in progenitors number and formation of cortical folds		Chenn and Walsh (2003)
*Pard3* (Par3)	E12 mouse RNAi-mediated knockdown by *in utero* lentiviral injection	Perturbation in the apical Par polarity complex	Decreased progenitor proliferation; Premature cell cycle exit and neuronal differentiation		Costa et al. (2008)
*Pard6* (Par6)	E13 mouse overexpression by *in utero* retroviral injection	Increased progenitor number	Prolonged maintenance of VZ progenitors		Costa et al. (2008)
*Cdc42* (Cdc42)	*Emx1-Cre* conditional knockout mouse	Gradual disappearance of adherens junctions; Detachment of RGCs from the ventricular surface and conversion into basal progenitors	Severely disorganized cortex; Increased cortical thickness due to increased neurogenesis		Cappello et al. (2006)
*Numb* (Numb) and *Numbl* (Numbl)	*Nestin-Cre Numb* and *Numbl* double conditional knockout mouse	Loss of progenitor radial organization	RGC clustering in neurogenic foci along the cortex; Increased progenitor number; Increased apoptosis; Reduced neuronal differentiation; Disruption of neuroepithelium integrity		Petersen et al. (2002, 2004), Li et al. (2003)
*Aspp2* (Aspp2)	Knockout mouse	Disruption of both tight and adherens junctions; Progenitor expansion and mislocalization	Hydrocephalus; Drastic ventricular dilatation		Sottocornola et al. (2010)
*Lgl1* (Lgl1)	Knockout mouse	Disruption of apical tight and adherens junctions; Progenitor expansion; Loss of RGC polarity; Disruption of RGC apical domain	Hydrocephalus; Disruption of the VZ surface; Reduced differentiated neuronal cell population		Klezovitch et al. (2004)
*aPKCλ* (aPKCλ)	*Nestin-Cre* conditional knockout mouse	Loosely packed RGCs; Disrupted adherens junctions and apical processes	Rough ventricular surface at E15.5; VZ, SV and IZ severely disorganized and difficult to distinguish at E16.5		Imai et al. (2006)
*Mcph1* (Mcph1)	Knockout mouse	Spindle misorientation favoring asymmetric divisions; Delayed and imbalanced centrosomal maturation; Abnormal spindles and chromosome misalignment; Lengthening of cell cycle progression	Small brain due to 20% decrease in thickness and lateral dimensions; Decrease in progenitor proliferation and premature neurogenesis	Autosomal recessive primary microcephaly type 1	Gruber et al. (2011)
*Cdk5rap2* (Cdk5Rap2)	Homozygous mouse mutant	Mitotic delay; Aneupolar spindle poles; Spindle orientation defects	Decreased cortical thickness mainly in superficial layers; Decrease in the size of apical and basal progenitor populations; Progenitor and neuronal cell death	Autosomal recessive primary microcephaly	Lizarraga et al. (2010)
*Cenpj* (Cenpj)	*Nestin-Cre* conditional knockout mouse	Mitotic delay and cell death of delocalized progenitors	Decreased brain size and cortical thickness	Autosomal recessive primary microcephaly	Insolera et al. (2014)
*Plk4* (Plk4)	*Nestin-Cre* conditional overexpressing mouse	Supranumerary centrosomes; Multipolar spindles; Prolonged mitosis; Frequent aneuploidy and apoptosis	Drastically reduced brain size; Decreased radial thickness; Reduced numbers of apical progenitors, basal progenitors and post-mitotic neurons	Microcephaly, growth failure and retinopathy	Marthiens et al. (2013), Martin et al. (2014)
*Htt* (Huntingtin)	E14.5 mouse RNAi-mediated knockdown by *in utero* electroporation	Spindle orientation defects	Increased neuronal differentiation at the expense of progenitors		Godin et al. (2010)
	*Nestin-Cre* conditional knockout mouse	Spindle orientation defects	Increased neuronal differentiation at the expense of progenitors		Godin et al. (2010)
	Homozygous mutant huntingtin (glutamine expansion) carrying mouse	Spindle orientation defects	Thinner VZ and thicker CP; Thinner total cortical thickness		Molina-Calavita et al. (2014)
*Tcof1* (Treacle)	Heterozygous mouse	Spindle orientation defects; M-phase extension and mitotic delay	Brain hypoplasia; Reduced number of neurons; Reduced apical progenitor population	Treacher Collins Syndrome (TCS) showing microcephaly	Sakai et al. (2012)
*Nde1* (Nde1)	Knockout mouse	Mitotic spindle defects resulting in mitotic delay/arrest; Increased horizontal cleavage orientation	Small-brain phenotype; Thinning of the cortex more pronounced in superficial cortical layers	Micro-lissencephaly syndrome due to defects in neuron production and cortical lamination	Feng and Walsh (2004)
*Nde1, Lis1* (Nde1, Lis1)	*Nde1* null and *Lis1* heterozygous double mutant mouse	Decreased self renewal and accelerated cell cycle exit; Increase in horizontal mitosis; Defects in metaphase plate formation; Failed mitotic spindle function; Disruption of apical	Severely disorganized and thinner cerebral cortex; Lack of distinct cellular layers; Reduced radial unit number	Micro-lissencephaly	Pawlisz et al. (2008)
		integrity and lateral contacts during mitosis
*Lis1* (Lis1)	*hGFAP-Cre* conditional knockout mouse	Spindle orientation defects; Premature reduction of RGC population	Thinner cortex; Less organized cellular structure	Lissencephaly	Yingling et al. (2008)
*PP4c* (PP4C)	*Emx1-Cre* conditional knockout mouse	Spindle orientation defects; Premature reduction of RGC population	Thinner and disorganized cortical layers		Xie et al. (2013)
*Vangl2* (Vangl2)	Knockout mouse	Spindle orientation defects	Reduced size of the cortex; Premature progenitor differentiation; Decreased neuronal production		Lake and Sokol (2009)
*Insc* (Insc)	*Nestin-Cre* conditional knockout mouse	Spindle orientation defects	Thinner cerebral cortex; Decreased neurogenesis		Postiglione et al. (2011)
	Ubiquitous overexpression in mouse	Spindle orientation defects	Thicker cerebral cortex; Increase in basal progenitor number; Increased neurogenesis		Postiglione et al. (2011)
*Wnt3a* (Wnt3a)	E13.5 overexpression by *in utero* electroporation in mouse	Progenitor expansion	Increased VZ thickness; Increased basal progenitor number and differentiation into neurons; Neuronal heterotopia; Thinner cerebral cortex		Munji et al. (2011)
*Pax-6* (Pax-6)	Homozygous loss of function mouse	Altered spindle orientation; Unequal inheritance of apical membrane domains; Decrease in apical complex proteins transcription	Decreased tangential expansion of the cerebral cortex; Ectopic progenitors		Asami et al. (2011)
*LGN* (LGN)	Knockout mouse	Randomized mitotic orientation	Decreased thickness of the VZ; Ectopic Pax6+ and Tbr2+ progenitors		Konno et al. (2008)
*Eml1* (Eml1)	Homozygous loss of function mouse	Defects in mitotic spindle orientation	Slightly decrease in VZ thickness; Both Pax6+ detached and Tbr2+ ectopic progenitors	Periventricular and globular ribbon-like subcortical heterotopia; Macrocephaly; Hydrocephaly	Kielar et al. (2014)
*Kif20b* (Kif20b)	Homozygous loss of function mouse	Abnormally shaped and misaligned midbodies	Reduced cortical thickness; Greatly reduced output progeny of apical progenitors		Janisch et al. (2013)

Microcephaly (e.g., OMIM 251200, 605481, Gilmore and Walsh, 2013) in human refers to a disorder in which the brain at birth is found to be significantly (−2.5–3 standard deviations below the mean) smaller than control brains. This condition leads to intellectual disability. In microcephalia vera, or primary microcephaly, although the brain is proportionally smaller, brain architecture seems not to be dramatically changed and the brain exhibits cortical folds. Its small size is indicative of a highly reduced number of neurons, premature neurogenesis or excessive cell death is likely, and most of the genes identified suggest roles in centrosomal-associated activities during division (see Table 1 and Section RGCs and Cell Division, Mechanisms Leading to Microcephaly, Gilmore and Walsh, 2013). There are a number of related disorders with microcephaly and additional cortical malformations, such as microcephaly with a simplified gyral pattern (MSGP, Adachi et al., 2011), or complex cortical malformations and polymicrogyria (e.g., OMIM 603802, 604317, e.g., Bilgüvar et al., 2010; Yu et al., 2010). These less “pure” forms show, as well as a reduced brain size, more noticeably affected gyrations (simplified or multiple small gyri), implying parallel changes in neuron production, organization and brain architecture. There is now known to be overlap between “pure” forms and those more obviously affecting gyri, as shown by unbiased genetic studies revealing mutations in genes previously identified mutated in other variants of the pathology (Poulton et al., 2014). Whole exome or genome sequencing is extremely useful in this respect, revealing unexpected genes associated with wider phenotypes than initially thought.

Macrocephaly (e.g., OMIM 600302) potentially has multiple origins related either to increased neuron number (inverse situation compared to microcephaly) but also to increased neuropil (e.g., dendritic arborizations), the latter linked to conditions such as autism spectrum disorder (OMIM 605309). Macrocephaly due to increased neuron number is not yet as clearly elucidated as conditions such as microcephaly, probably related to the multiple potential causes of this disorder, and different types of progenitors found in primate, which are not easily studied in the rodent (Hansen et al., 2010; Wang et al., 2011). Future studies with human genetics as a starting point (and see e.g., Keeney et al., 2014a,b) will almost certainly shed further light on this condition.

## Mouse and human mutations and mechanisms important for RGC function

### General characteristics of RGCs

One characteristic aspect of RGCs is their intrinsic highly polarized structure with the cell body confined to the ventricular zone (VZ), and two processes that depart from it: a long basal process reaching the pial surface, and a short apical process descending to the ventricular lining. RGCs need both processes to exert their function: the basal process constitutes the scaffold for migration of newly born neurons through the intermediate zone (IZ), while the apical process is responsible for attachment to the ventricular lining and contains key elements of signaling pathways. These are important to control the balance between proliferation and differentiation, and for cellular specification. RGCs, which are Pax6-positive, as well as self-renewing, can give rise to basal progenitors in the subventricular zone (SVZ) which are Tbr2-positive, these then give rise to post-mitotic neurons. These and further progenitor types are greatly expanded in the primate cortex (reviewed by LaMonica et al., 2012).

**Figure 2:**
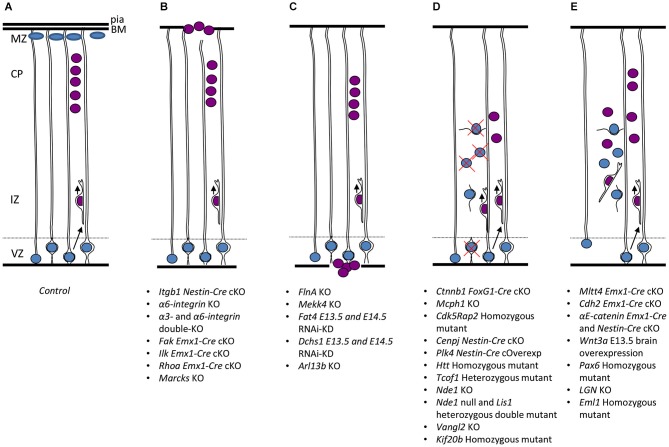
**RGC mechanisms leading to mouse malformations. (A)** Control situation, apical progenitors (containing blue nuclei) divide by interkinetic nuclear migration (INM) in the VZ, neurons (burgundy nuclei) migrate radially on RGC basal processes across the IZ, to settle in the CP. Cajal Retzius cells (ovals) present in the MZ secrete signals to the migrating neurons. End-feet of RGC basal processes receive signals from ECM molecules in the BM close to the pial surface. **(B)** Cobblestone lissencephaly phenotype, in this case some RGC basal processes are not well attached to the pial surface, possible breaks in the BM potentially cause neurons (burgundy nuclei at the surface of the brain) in some regions to overmigrate into the meningeal space. **(C)** Periventricular disorganization, some neurons (burgundy nuclei) remain stuck at the ventricular surface, most probably due to breaks in the ventricular lining where apical end-feet of RGCs normally attach. **(D)** Microcephaly phenotype, two potential mechanisms may give rise to this malformation leading to a greatly reduced size of the brain. Some mouse models suggest that premature differentiation of progenitors into post-mitotic neurons (burgundy nuclei within radially migrating neuron close to VZ) depletes the progenitor pool (represented by red cross over blue nuclei in VZ). Other studies show instead increased cell death of abnormal progenitors (red cross over blue nuclei present in IZ). **(E)** Globular heterotopia (e.g., *HeCo* mice), in this case a proportion of apical progenitors detach from the ventricular surface (represented by blue nuclei without apical attachment to the ventricular lining) and retain proliferation capacity, providing a local source of neurons in the IZ (burgundy nuclei). A subcortical heterotopia subsequently arises. VZ, ventricular zone; IZ, intermediate zone; CP, cortical plate; MZ, marginal zone; BM, basement membrane.

**Figure 3:**
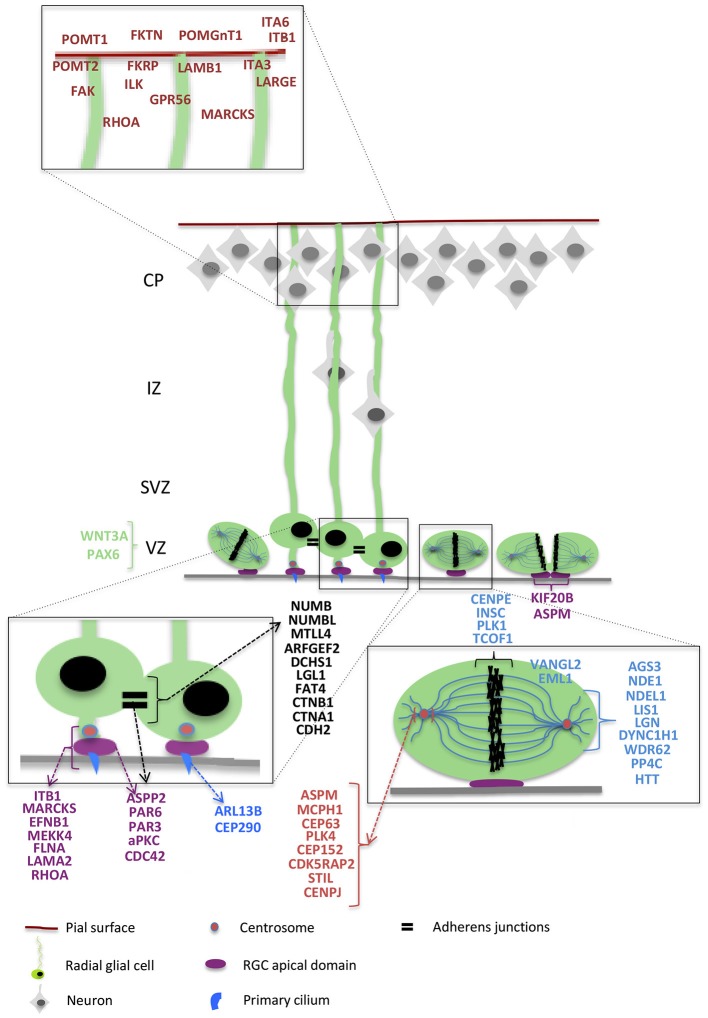
**Radial glial cell and different genes**. Schematic representation of interphase and dividing RGCs (in green) and neurons (in gray) migrating along basal RGC processes. Higher magnifications show RGC structural details such as basal attachment to the pial surface (represented in red), apically located adherens junctions (black), apical attachments and midbody (purple), centrosomes (pink), primary cilia (blue), and a mitotic cell with the MTs organized in the mitotic spindle (light blue) and the DNA aligned at the metaphase plate (black). The ventricular surface is represented as a gray line. The different genes are represented close to the structure in which they have clearly been shown to be involved based on the classification proposed in this review, with a color corresponding to the color of the structure. Genes involved in RGC basal process attachment to the pial surface are represented in red; in black are genes linked with adherens junctions; in purple are genes participating in apical polarity, attachment to the ventricular surface, and in the apically-located midbody; in blue are genes essential for the primary cilium; in pink are represented centrosome-related genes, and in light blue genes participating in the regulation of mitotic spindle function. Genes that are involved in transcriptional regulation are represented in green.

As RGCs present a very specialized morphology and dynamics, which are strictly linked with the function they exert during cortex development, every minor perturbation involving their structure or behavior is susceptible to lead to major developmental problems. Indeed, numerous genes coding for proteins influencing RGC morphology and function have been found mutated in mouse models and in cortical malformation patients. We try to bring together here mouse mutant data related to these genes (also resumed in Table 2), classifying these data by different RGC compartments and cellular mechanisms (resumed in Figures 2, 3).

### Basal processes of RGCs, shedding light on heterotopias, polymicrogyria and type II lissencephaly

Perturbations that affect basal process structure can cause subsequent problems of neuron migration and lead to cortical malformations such as heterotopia or cobblestone lissencephaly. Breaks of the cortical basement membrane (BM; Figure 2) have been associated with RGC basal process end-feet that are not well attached to the extracellular matrix (ECM). The meningeal BM is located immediately below the pia matter and serves as an anchor point for the end-feet of RGCs and as a physical barrier to migrating neurons. Through human genetics studies, it has been shown that cobblestone lissencephalies are associated with reduced glycosylation of alpha-dystroglycan, a basal process dystrophin-associated glycoprotein that is crucial to act as anchor between the dystrophin complex and the ECM (e.g., laminin, van Reeuwijk et al., 2005; Roscioli et al., 2012; Buysse et al., 2013). Six major genes have been identified encoding putative or demonstrated glycosyltransferases, POMT1, POMT2, FKTN, FKRP, LARGE and POMGnT1. Post-mortem studies have helped characterize this disorder, however, mouse models for these genes are often not viable, which has led to the difficulty of studying the exact mechanisms involved (Brockington et al., 2001; Yoshida et al., 2001; Beltrán-Valero de Bernabé et al., 2002; Longman et al., 2003; van Reeuwijk et al., 2005; Manzini et al., 2012; Roscioli et al., 2012; Willer et al., 2012). Fragmentation of the BM is however, also frequently seen after deletion of other ECM components or receptors. Laminins are major secreted glycoproteins found in the BM, where they influence cell proliferation, differentiation, migration, and adhesion. *LAMB1*, encoding the laminin subunit beta-1 is involved in basal process attachment to the pial surface and also found mutated in a cobblestone brain malformation (Radmanesh et al., 2013). Thus, mutations in laminin subunit genes, as well as glycosyltransferases, may both lead to detachment of RGC processes from the pial surface leading to breaches of the BM, disintegration of the scaffold mediating neuronal migration, subcortical heterotopias and neuronal overmigration phenotypes. These studies hence reveal the progenitor origin of certain “neuronal migration” defects (but see also Moers et al., 2008, for intrinsic problems in migrating neurons, affecting potentially their ability to stop migrating).

Similarly, the *GPR56* gene encodes a heterotrimeric guanine nucleotide-binding adhesion protein (G-protein)-coupled receptor that is highly expressed in progenitors, is localized to the basal process, and binds ECM proteins at the pial surface (Li et al., 2008; Luo et al., 2011). Disruption of *GPR56* was found to selectively and bilaterally perturb the human cortex surrounding the Sylvian fissure with a strikingly restricted polymicrogyria (Bae et al., 2014). Loss of *GPR56* disrupts RGCs pial anchorage and causes breaks in the BM, through which some neurons over-migrate (Li et al., 2008; Bahi-Buisson et al., 2010). Studies involving this gene hence link these phenotypes to mechanisms leading to polymicrogyria. Moreover, even when the pia is intact, as observed in *Gpr56* knockout mice, cortical thickness and organization are irregular with periodic thinner regions. Such defects suggest proliferation problems and indeed in these mice there are less mitotic progenitors in both the VZ and SVZ at embryonic day (E) 14.5. Conversely, in mice carrying a transgene that overexpresses human *GPR56*, the opposite effect was observed (Bae et al., 2014). These data show that disruption of basal processes and overmigration, can be intimately linked with a perturbation of proliferation.

RGC basal processes have been postulated to regulate progenitor proliferation via integrin signaling (Radakovits et al., 2009; Fietz et al., 2010, see Section Apical Adhesive Interactions and Mechano-Transduction, Shedding Light on PH and Ciliopathies). *GPR56* influences attachment to ECM proteins, such as collagen type III, and tetraspanins, which are known to also bind integrins expressed by basal end-feet (Xu and Hynes, 2007; Li et al., 2008; Luo et al., 2011). Studies in conditional β1-integrin knockout mice showed a wavy appearance of cortical layers at E15.5, indicative of defects in the organized laminar cytoarchitecture and abnormal positioning of cortical neurons. Neurons either invaded the marginal zone (MZ) or accumulated deep in the cortical wall, resembling cobblestone lissencephaly. RGC fibers in the mutants terminated at varying positions close to the BM and were highly irregular (Graus-Porta et al., 2001). All these observations, together with the finding, by conditional ablation specifically in neurons, that β1-integrins are not essential for neuron-glia interactions and neuronal migration *per se* (Belvindrah et al., 2007), indicated that they are likely to primarily be required for anchorage to the BM. Defects similar to those found in β1-integrin knockout mice were also found in mice with mutations in the genes encoding the integrin α6-subunit or both α3 and α6 (Georges-Labouesse et al., 1998; De Arcangelis et al., 1999; Hynes, 2002); other components of the BM (Miner et al., 1998; Halfter et al., 2002; Pöschl et al., 2004); and the integrin downstream effectors focal adhesion kinase (FAK; Beggs et al., 2003) and integrin-linked kinase (ILK; Niewmierzycka et al., 2005).

Studies involving β1-integrin, the small GTPase RhoA and the protein Marcks (Myristoylated alanine-rich substrate protein) also highlight the fact that a number of proteins are likely to have a role in both apical and basal processes. The conditional deletion of RhoA and Marcks in the developing mouse cortex leads to a prominent tissue mass (heterotopia) found underneath an apparently layered but thinner cortex. Moreover, in the case of RhoA deletion, there was a phenotype reminiscent of cobblestone lissencephaly. Analysis of progenitor morphology also revealed already at E12.5, that mitotic cells were scattered in the cortex instead of being neatly aligned at the ventricular lining. At E16 mitotic cells were assembled into a broad band located abnormally in the middle of the cortex between the pial and the ventricular surfaces. Moreover, RGCs had mis-oriented processes and had lost their apical anchoring. While RhoA-depleted neurons still migrated fairly normally in a wild-type environment, they followed a largely non-radial path when the RGC scaffold was disturbed by RhoA depletion (Cappello et al., 2012). RhoA plays a role in polymerizing actin into fibers (F-actin) (Etienne-Manneville and Hall, 2002). Thus, loss of RhoA destabilized the actin and microtubule (MT) cytoskeletons in both neurons and RGCs but the most severe consequences were in RGC positioning and in the proper formation of the basal scaffold (Cappello et al., 2012). Knockout mice for Marcks, an actin-cross-linking protein and subcellular substrate of protein kinase C (PKC), also presented a disorganized RGC scaffold, impaired cell polarity, disorganized VZ, and ectopic progenitors. Marcks is a potent upstream regulator of the localization and function of cell polarity complexes, and its mutation leads to disrupted VZ organization and mitotic orientation (Blackshear et al., 1997; Weimer et al., 2009). At E15.5, the RGC scaffold was severely disrupted and basal end-feet failed to branch appropriately and instead had a club-like, balled-up appearance as they reached the pial surface. There was a reduction in the thickness of both the VZ and SVZ, and perturbed migration. Phenotypes combining apical and basal perturbation thus show misplaced progenitors, perturbed proliferation and either inability of neurons to reach the cortex or overmigration. In the different models whether the basal detachment causes the detachment of the apical process, or *vice versa*, is not always clear. These studies also emphasize that the cytoskeleton is essential for the maintenance of RGC structure.

### Apical adhesive interactions and mechano-transduction, shedding light on PH and ciliopathies

Perturbations of the apical domain of RGCs, a complex and relatively well-studied cell compartment, are becoming recognized as leading to neuroepithelial disorganization, different types of heterotopia (this section), or micro- and macro-cephaly (Section RGCs and Cell Division, Mechanisms Leading to Microcephaly), depending on the affected cell mechanisms. The consequence of the perturbation can appear as obvious breaks in the ventricular lining and changes in VZ architecture, or be more subtle. To help explore these phenotypes, we further focus in this section on molecules which have been described to play a role in intercellular and ECM contacts in the VZ.

We re-mention here β1-integrins which have also been shown to be involved in end-feet anchoring to the ventricular surface, binding with laminins, located also in the apical ECM. These anchors are reinforced by cadherin–catenin-based adherens junctions, which help attach apical end-feet of adjacent RGCs to each other (Kadowaki et al., 2007). Blocking β1-integrin’s function by injection of specific antibodies into the lateral ventricles of embryos at E12.5 and E15.5 showed a significant increase in dividing cells due to a larger abventricular (localized outside the VZ) dividing progenitor population (Loulier et al., 2009). This phenotype was linked to the detachment of apical processes from the ventricular surface and alterations in mitotic spindle orientation showing that β1-integrin plays a critical role in the adhesion that maintains the progenitor cells within their niche and preserves the architecture of the VZ. The same effect was observed in laminin-α2 deficient mice (Miyagoe et al., 1997; Loulier et al., 2009). Another link between apical VZ integrity and heterotopia formation is represented by the conditional deletion in the cortex of the apical junction molecule α-E-catenin. Due to the disruption of RGC morphology, caused by impaired actin cytoskeletal organization, progenitors were found disorganized in rosette-like structures, associated with a large heterotopia and a thin layered cortex (Schmid et al., 2014). Another component of adherens junctions is Afadin, which interacts with cadherins and stabilizes them (Sato et al., 2006). Its conditional inactivation in the developing cortex leads to disruption of adherens junctions, dispersion of dividing progenitors with a shorter cell cycle and reduced cell cycle exit, and formation of a double cortex-like phenotype (Gil-Sanz et al., 2014). In the same study, conditional knockout mice for a cadherin subunit, *Cdh2*, showed a very similar phenotype, close also to that described previously for RhoA-knockout mice (Cappello et al., 2012). Thus intercellular contacts and downstream pathways seem to be essential to maintain the integrity of the VZ and to regulate proliferation.

Similarly, the Eph/ephrin signaling pathway (Nievergall et al., 2012) activates signal transduction cascades, and exhibits extensive cross-talk with other receptors, including cadherins and integrins (Arvanitis and Davy, 2008). Ephrin B1 is expressed in apical progenitors from the neuroepithelial stage in a ventricular-high to pial-low gradient (Stuckmann et al., 2001). Prolonged Ephrin B1 activity was shown to prevent progenitor differentiation, while loss of function had an opposite effect promoting differentiation and leading to loss of progenitor cells (Qiu et al., 2008; Murai et al., 2010). Indeed, Ephrin B1 reverse signaling controls the switch between progenitor maintenance and neuronal differentiation (Arvanitis et al., 2010). In knockout mice, and more severely in heterozygous mice, the neuroepithelium had an irregular appearance with formation of micro-invaginations due to abnormal folding of the VZ without changes in apico-basal polarity of progenitors. Progenitor detachment was also observed. In absence of Ephrin B1, local alterations of the apical surface might weaken the rigidity and cohesion of the neuroepithelium. The reason why in heterozygous embryos the phenotype is more severe might be that sorting between Ephrin B1-positive and Ephrin B1-negative cells leads to discontinuous rigidity, which is more detrimental to the morphogenesis of this tissue than a homogeneous decrease in rigidity. Thus, the normal function of Ephrin B1, via an interaction with EphB2 on neighboring cells, is to maintain morphology and localization of progenitors in the VZ by promoting apical integrin-based adhesion (Arvanitis et al., 2013).

Mutations in the Filamin A (*FLNA)* gene were found in 100% of families with X-linked bilateral PH and in 26% of sporadic patients with PH (Fox et al., 1998; Parrini et al., 2006). β1-integrin mediated adhesion to the ECM was also found to be dependent on the binding of FLNA to vimentin and PKC epsilon (PKC1, Kim et al., 2010) allowing vimentin phosphorylation by PKC1. This step is crucial for the activation and trafficking of β1-integrin to the plasma membrane. *FLNA* encodes a large phosphoprotein that crosslinks actin filaments into orthogonal networks, reorganizing them by interacting with several proteins at the membrane (Stossel et al., 2001; Nakamura et al., 2007). It may play a role both in progenitors and migrating neurons. The ventricular surface has been shown to be disrupted in FlnA knockout mice (Feng et al., 2006). PH formation and alterations in the neuroepithelial lining were also shown in FlnA-knockdown brains where disruption of both the polarized RGC scaffold and the neuroepithelial lining were the likely cause of the PH (Carabalona et al., 2012). Also, loss of mitogen-activated protein kinase kinase kinase 4 (MEKK4) in mice, a regulator of FlnA phosphorylation, leads to a similar phenotype (Sarkisian et al., 2006). A second human PH gene, *ARFGEF2*, coding for brefeldin-A-inhibited guanine exchange factor-2 (BIG2) is likely to play a role in endocytosis, regulating levels of Arf1 at the plasma membrane, which is known to regulate cell-cell contacts (Zhang et al., 2013). PH was also induced by knockdown of C6orf70 in the developing rat cortex, a gene of unknown function, mutated in a PH patient, and coding for a protein with a vesicle-like subcellular localization (Conti et al., 2013). These combined data suggest a coordinated role for actin and vesicle trafficking in controlling cell adhesion in apical regions in the VZ (Sheen, 2014).

Related to this, protocadherins Dchs1 and Fat4 (Cappello et al., 2013) are important for an apically located adhesive complex (Ishiuchi et al., 2009). Van Maldergem syndrome, an autosomal-recessive multiple malformation syndrome, shows a partially penetrant PH phenotype caused by mutations in *FAT4* or *DCHS1*. Dchs1 is the ligand of the Fat4 receptor and the complex they constitute is situated apically, closer to the ventricle relative to adherens junctions. Fat4 and Dchs1 knockdown studies in mice also showed an increased cell proliferation in the VZ and SVZ, a block in differentiation between the Pax6+ and Tbr2+ states, and an accumulation of neuronal precursors, showing that this adhesive complex normally suppresses continued proliferation (Cappello et al., 2013). Adhesion and proliferation hence seem to be interlinked themes related to these phenotypes.

Another very important characteristic of the apical domain of RGCs is the presence of the primary cilium, an MT-based, slender projection from the cell that is thought to be important for sensing signaling factors present in the cerebrospinal fluid, and with a guiding role in the establishment of apical-basal polarity of the RGC scaffold. The importance of cilia function for cortical development is evident in developmental brain disorders such as Joubert, Meckel-Gruber, orofaciodigital and Bardet-Biedl syndromes (commonly referred to as ciliopathies), where disrupted cilia and the resulting changes in cortical formation may underlie cognitive deficits and intellectual disability (Cantagrel et al., 2008). Mutations in a gene encoding the centrosome-associated protein CEP290, important for ciliogenesis (Kim et al., 2008), have been found in both Meckel-Gruber and Joubert syndromes (Valente et al., 2006; Frank et al., 2008). Also, Arl13b, a small GTPase of the Arf/Arl family that is mutated in Joubert syndrome, is specifically localized to cilia and controls the MT-based, ciliary axoneme structure. Deletion of Arl13b impairs the cilium’s ability to convey critical extracellular signals such as Shh (Caspary et al., 2007). In constitutive mutant mice, and in E9 conditional knockout mice, early neuroepithelial progenitors showed markedly perturbed polarity with the soma located near the pial surface and the basal end-feet located near the VZ. These cells divided ectopically at or near the pial surface, instead of adjacent to the ventricular surface (Higginbotham et al., 2013). These studies show that primary cilia play an important role in both signal transduction and polarity.

Indeed, RGC polarity is a crucial issue for cortical development. We resume this only briefly here (see details in Table 2 and Figure 3). Conditional mutagenesis in the mouse or focal knockdown experiments, have often been necessary to reveal the role of a particular polarity protein, in this case it remains difficult to directly link these data with malformations. What clearly comes out of the different studies is the relationship between apical polarity complexes (Par-Complex and its regulators), maintenance of the structure of the ventricular lining and neuroepithelium, and regulation of cell proliferation/differentiation and fate. Thus, defects in the polarity complexes have been studied both prior to neurogenesis and during the neurogenic period. Changes in the balance between expansion of RGCs, production of basal progenitors, and differentiation of post-mitotic neurons have been identified but are still little-understood. This imbalance can be the cause of the incapacity of the brain to form an ordered layer structure and/or a brain of the correct size (see also Section RGCs and Cell Division, Mechanisms Leading to Microcephaly). Diverse mechanisms can be affected by the perturbation of different polarity molecules, related to the complex interactions that link the different players. The variable consequences are also likely to be due to the different importance these molecules have during neuroepithelial progenitor expansion and/or the neurogenic phase when RGCs have a major role. Also, cell adhesion complexes, strictly related to polarity components, change during the transition from early neuroepithelial cells to RGCs (Götz and Huttner, 2005), adding to the complexity. Mechanisms leading to hydrocephaly identified in some mutant mice remain complex, however, disruption of the early neuroepithelium and polarity changes are clearly associated.

### RGCs and cell division, mechanisms leading to microcephaly

We previously discussed how cell junctions and the integrity of apical polarity domains are important for regulating the structure of the ventricular lining and the balance between proliferation and differentiation. However, there are other cellular mechanisms that are more solely linked with proliferation/differentiation and cell fate. RGC centrosome behavior, mitosis, the regulation of spindle orientation (which has effects on symmetric and asymmetric division and cell localization), cytokinesis and interkinetic nuclear migration (INM) are finely regulated processes, and several cortical malformation genes or mouse mutants associated with these mechanisms have been studied. We have classified these phenotypes within separate sub-categories, but these can still often be considered as overlapping.

#### Microcephaly genes and centrosome function

The centrosome is important for correct spindle assembly and function during mitosis. Centrosomes influence the morphology of the MT cytoskeleton, function as the base for the primary cilium and integrate important signaling pathways. At the core of a typical centrosome are two cylindrical MT-based structures termed centrioles, which recruit a matrix of associated pericentriolar material (Nigg and Stearns, 2011). RGC centrosomes are located at the extremity of the apical process and are aligned at the ventricular surface. This position influences cell polarity and anchoring in the VZ. Once a cell enters mitosis, centrosome duplication takes place and these move more basally to help form the spindle poles and the bipolar mitotic spindle. A set of proteins related to centrosome behavior has been identified, whose mutation was found to cause microcephaly. Mutations in ASPM (abnormal spindle-like microcephaly associated) are the most common cause of primary microcephaly in humans (Kumar et al., 2004; Pichon et al., 2004; Shen et al., 2005; Gul et al., 2006). Aspm has been shown to exert a critical role at the spindle poles of neuroepithelial cells, maintaining spindle position during mitosis and, consequently regulating the precise cleavage plane orientation required for symmetric, proliferative divisions (Fish et al., 2006). Microcephalin (*MCPH1*) mutations also cause primary microcephaly type 1 (Woods et al., 2005). *Mcph1* is expressed at high levels in the VZ and SVZ at E13.5 and E15.5 (Gruber et al., 2011) and *Mcph1*-deficient mice have a small brain. Characterization of the mutant cortex revealed premature production of neurons and exhaustion of progenitors. Mcph1 deficiency specifically caused a delayed and imbalanced centrosomal maturation, leading to a lengthening of the cell cycle due to abnormal spindles and chromosome misalignment (Gruber et al., 2011). Another example of a gene mutated in primary microcephaly is SCL-interrupting locus protein (*STIL*), encoding a centriole-duplication factor that localizes to the procentriolar cartwheel region, a key structure in procentriole assembly. STIL depletion was shown to completely block centriole formation, whereas its overexpression resulted in extensive centriole amplification (Arquint and Nigg, 2014).

Human primary microcephaly is also caused by mutations in *CDK5RAP2* (cyclin-dependent kinase 5 related activator protein 2, Bond et al., 2005). In somatic cells, CDK5RAP2 promotes centrosomal cohesion (Graser et al., 2007) and recruits the γ-tubulin ring complex (γ-TuRC)—the MT nucleator—to the centrosome (Fong et al., 2008). In a homozygous mouse model of *Hertwig’s anemia (an)*, the disease is caused by a mutation in *Cdk5rap2* (Lizarraga et al., 2010). Brain size was reduced and an increased ventricular size and decreased cortical thickness were already detected at E13.5. Mutant animals had fewer total neurons and the last-born superficial neurons were particularly reduced. The premature decrease in progenitors was due to problems encountered during mitosis causing cell death affecting both progenitors and neurons and, possibly, changes in cell fate. Indeed, an increase in pro-metaphase and metaphase precursor cells with mono-, tri-, and other aneupolar spindle poles, together with defective spindle orientations, were detected (Lizarraga et al., 2010). Mutations in centromere protein J (CENPJ) also cause microcephaly (Bond et al., 2005). This gene is involved in the maintenance of centrosome and spindle integrity. A recent study described conditionally inactivated Cenpj also known as SAS-4 (Insolera et al., 2014). This led to mitotic delay, p53 activation and cell death of delocalized progenitors. Keeping cells alive by p53 inactivation showed many RGCs in the IZ, which were multipolar but could still divide, self-renewing and producing also basal progenitors and neurons. Under these conditions small heterotopias formed in the IZ. This study showed that cell death was not due to aneuploidy or other chromosomal abnormalities, unlike hypomorphic Cenpj mutants (McIntyre et al., 2012), instead delocalized RGCs were often remarkably deficient in centrioles and cell death was most probably due to mitotic delay.

Centrosome amplification may also cause microcephaly by affecting the correct formation of the spindle and continuation through mitosis. Polo-like kinase 4 (Plk4) is a centriole duplication protein whose overexpression leads to cells with supernumerary centrosomes. Conditional overexpression of Plk4 specifically in progenitors, led to reduced brain size, accompanied by a reduction of both apical and basal progenitors, and the neuronal population. In Plk4 overexpressing embryos, cells with extra centrosomes showed bipolar, as well as multipolar, spindle configurations, and spent more time in mitosis. This was at the origin of p53-dependent cell death and could be one of the main causes of brain reduction in this model. Deletion of p53 showed accumulation of aneuploid daughter progenitors, and these underwent premature neuronal differentiation, with subsequent depletion of the progenitor population (Marthiens et al., 2013). Related to this work, another two MCPH proteins, CEP63 and CEP152, form a complex that is an essential part of the molecular machinery controlling centrosome numbers, and defects in either component result in a diminished pool of precursors that cannot provide an adequate supply of neurons (Sir et al., 2011). Thus centrosome formation, numbers, maturation and function are all important for maintaining a correct progenitor number.

#### Spindle genes and mitosis

The cell cycle of RGCs is characterized by an oscillatory behavior called INM. Mitosis occurs apically close to the ventricular surface, nuclei then migrate basally during G1 to reach the most basal side of the VZ where they undergo S-phase, and migrate apically during G2 to reach the ventricular surface before undergoing mitosis again. This behavior of the nuclei of RGCs gives the VZ the appearance of a pseudo-stratified epithelium. A variety of molecules (Kif1a, Dynein (Tsai et al., 2010); Lis1 (Cappello et al., 2011); Tag-1 (Okamoto et al., 2013); Rnd3 (Pacary et al., 2013); Dock7, Tacc3 (Yang et al., 2012); SUN-KASH protein complex (Zhang et al., 2009); Tpx2 (Kosodo et al., 2011)) have been reported to play a role in this process, although since no cortical malformation in human has been shown to our knowledge to be caused directly by abnormal INM (but see discussion below for dynein and LIS1, and Asp in *Drosophila* (Rujano et al., 2013)), we do not mention them further here. We focus instead on mitosis itself and division occurring at the ventricular lining.

Even if the mechanisms of mitosis are still not clear, the orientation of the mitotic spindle was previously linked with symmetric or asymmetric modes of cell division and, consequently, also with the progenitor state or cell cycle exit. This remains a little-understood area. Mitotic division planes are coordinated with the polarized expression of cell fate determinants such as Numb, β-catenin, Par3 and Notch (Zhong et al., 1996; Chenn and Walsh, 2002; Bultje et al., 2009). In order to be RGCs, daughters of dividing progenitors need to inherit both the apical and the basal attachments, this is favored when the spindle is oriented parallel to the ventricular lining with a cleavage plane that bypasses both the apical and basal domains (Taverna et al., 2014). The molecular mechanisms that govern the mode of cell division in RGCs are still not clear (Knoblich, 2001; Lancaster and Knoblich, 2012). Orientations of the spindle other than parallel may favor asymmetric divisions and the generation of neurons or basal progenitors, which do not inherit the apical attachment, and migrate to the SVZ to undergo a symmetric final division and generate two neurons (Postiglione et al., 2011). However, other studies concern models in which ectopic RGC progenitors result from perturbations of spindle orientation and the unequal inheritance of apical attachment sites upon division, with the retention however, of the molecular signature of apical progenitors (Konno et al., 2008; review by Lancaster and Knoblich, 2012; Kielar et al., 2014). This suggests that the primary role of planar spindle orientation in apical divisions is to maintain daughter cells attached to the ventricular surface, but not directly to influence the choice between symmetric and asymmetric outcomes (Peyre and Morin, 2012). The size of the apical domain corresponds to only 1–2% of the total membrane surface. This is why minor changes in spindle orientation may decide whether the cleavage plane would dissect or bypass the small apical domain and result in its equal or unequal repartition and the distribution of cell fate determinants between the daughter cells (Kosodo et al., 2004; Marthiens and ffrench-Constant, 2009; Peyre and Morin, 2012). Moreover, defects in mitotic spindle assembly, dynamics and function have often been linked with mitotic delay, changes in cell cycle length and, consequently, of daughter cell fate. The cell cycle length of wild-type progenitor cells increases from 8.1 h at E11 to 18.4 h at E17 in mouse embryos. In contrast, the period of the G2/M-phase, is very rigidly controlled and remains constant at 2 h throughout brain development (Takahashi et al., 1995; Sakai et al., 2012). Therefore, altering M-phase progression is likely to influence cell survival and fate determination. Thus, even if the role of spindle orientation in cell fate and mode of division are not clear, its timely and mechanistic regulation are finely controlled processes, and mutations have been found in several genes which severely perturb the formation of the cortex, often causing different versions of microcephaly.

Interestingly, Huntingtin (Htt), the protein whose mutation leads to Huntington’s disease (HD), is one such gene. During mitosis, Htt was found specifically located at the spindle poles and at the spindle midzone (Godin et al., 2010). Htt was shown to control spindle orientation by ensuring the proper localization of several key components of the spindle and, as a consequence, its position. The MT-dependent transport of the dynein/dynactin complex to the spindle was reduced in Htt-depleted cells, and the localization of Protein Numa1 (NuMA) was modified. In mammalian cells, NuMA by assembling with dynein/dynactin is essential for the organization of MTs at the spindle pole (Merdes et al., 1996; Fant et al., 2004) and the regulation of astral MT interactions with the cell cortex (Du and Macara, 2004). Depletion of Htt by RNAi in progenitors *in vivo* led to spindle mis-orientation and promotion of premature neurogenesis (Godin et al., 2010; Molina-Calavita et al., 2014).

Spindle orientation is regulated by the interaction of astral MTs with the cellular membrane, and the polymerization of MTs directed toward the chromosomes assures their proper segregation. Related to this, mutations in the Treacher Collins Syndrome Treacle Protein (*TCOF1*) gene cause Treacher Collins Syndrome (TCS), which, amongst other defects, is associated with microcephaly. *TCOF1* codes for a nucleolar phosphoprotein known as Treacle (The Treacher Collins Syndrome Collaborative Group, 1996). *Tcof1* heterozygous mice exhibited considerable brain hypoplasia, with a reduced RGC population and cells already committed to neuronal fate. Vertical cleavage planes in dividing RGCs were found dramatically reduced showing that Treacle is important for correct spindle orientation. This was accompanied by an extension of M-phase and mitotic delay. Treacle was found to localize to centrosomes of RGCs during interphase and in mitotic cells, it co-localized with CENP-E at the kinetochore, and was also found at the midzone in anaphase cells and the midbody in telophase. In *Tcof1* knockdown cells, mitotic spindles were found disorganized, and chromosome assembly at the metaphase plate incomplete, suggesting roles for the Treacle protein in chromosome movement and spindle formation. Loss of Plk1 function, which phosphorylates Treacle, also resulted in perturbation of mitotic spindle orientation and mitotic delay (Sakai et al., 2012).

Thus, multiple human microcephaly proteins can take part in the assembly of the mitotic MT structure and its dynamics (Bond and Woods, 2006; Fish et al., 2006; Sun and Hevner, 2014; Valente et al., 2014). However, further similar function proteins seem also important for cortical layering. WD repeat-containing protein 62 (*WDR62*) encodes a centrosome- and spindle pole-associated protein in which mutations cause microcephaly with simplified gyri and abnormal cortical architecture (Bilgüvar et al., 2010; Yu et al., 2010). WDR62 accumulated strongly at the spindle poles during mitosis and the murine version, Wdr62 was found expressed in the neuroepithelium exclusively in apical precursors undergoing mitosis at the ventricular surface (Nicholas et al., 2010). Also, centromere-associated protein E (*CENPE*), the gene coding for centromere-associated protein E was found mutated in patients with microcephalic primordial dwarfism (MPD), featuring microcephaly and a simplified gyral pattern (OMIM 616051). Mutations in CENPE were shown to alter spindle dynamics and chromosome segregation leading to delayed mitotic progression (Mirzaa et al., 2014). CENPE is a core kinetochore component functioning initially to mediate the bringing together of misaligned chromosomes, and subsequently to capture spindle MTs during mitosis (Abrieu et al., 2000; Yao et al., 2000). Indeed, the stable propagation of genetic material during cell division depends on the congression of chromosomes to the spindle equator before the cell initiates anaphase (Kapoor et al., 2006). A replicated chromosome possesses two discrete, complex, dynamic, macromolecular assemblies, known as kinetochores that are positioned on opposite sides of the primary constriction of the chromosome. Proper chromosome congression depends on MT bundles (K fibers) that connect sister kinetochores of each chromosome to opposite spindle poles (Rieder and Salmon, 1998). CENPE is clearly involved in these processes, although the reason why layering is also affected with CENPE (or WDR62) mutations still remains unclear.

Similarly,* NDE1* is one of the known spindle-associated genes and mutations also cause a severe microlissencephaly syndrome that reflects both morphological and quantitative defects in RGCs. In apical cells, Nde1 was found enriched at the centrosome in interphase and early mitosis and then reduced during metaphase and telophase during which it was present at the mitotic spindle and at the level of kinetochores. NDE1 was shown to be important for normal mitotic spindle function (Alkuraya et al., 2011; Bakircioglu et al., 2011). Nde1 knockout mice showed a small-brain phenotype from birth (Feng and Walsh, 2004). The thinning of the cortex in these mice was much more pronounced in superficial cortical layers, which are formed near the end of neurogenesis. Mitotic spindle defects were described to result in mitotic delay/arrest and shifted orientation towards horizontal cleavage. Nde1 self-associates and has a scaffolding function in mitotic spindle assembly. Blocking its self-association induced defective centrosomal duplication, and this defect was at least partially responsible for observed spindle mis-assembly (Feng and Walsh, 2004). NDE1 is also a critical binding partner of LIS1 (Feng et al., 2000), a gene causative of neuronal migration defects and type I lissencephaly (Dobyns et al., 1993; Sicca et al., 2003). Nde1 null and Lis1 heterozygous double mutant mice showed not only a thinner but also a severely disorganized cortex where all the distinct cellular layers were lacking and reduced numbers of radial neuronal units were caused by loss of progenitors in early ages due to failed mitotic spindle function (Pawlisz et al., 2008). Lis1 is also a cytoplasmic scaffold protein that functions as an adaptor that controls the organization of the MT cytoskeleton and MT-associated motors, confirming their importance for spindle orientation (Faulkner et al., 2000; Yingling et al., 2008). Indeed, mutant RGCs were able to establish apical junctions and overall polarity, but failed to maintain apical cell shape and intimate association with the ventricular surface in particular during mitosis (Pawlisz et al., 2008). Thus, Nde1-Lis1 is essential for mitotic orientation determination, but also critically required for maintaining apical cell integrity and lateral contacts of RGCs during mitosis, showing that the polarity and morphology of metaphase progenitors must be co-regulated with mitotic spindle orientation for correct neuron number and organization (Pawlisz et al., 2008).

It is still unclear how LIS1 works in the human cortex, and if heterozygote gene dosage defects found in human lissencephaly patients affect more neuronal migration or progenitor proliferation. However, a role of Lis1 in spindle orientation was confirmed by studies in conditional knockout mice (Yingling et al., 2008). Complete Lis1 loss was found to have a deleterious effect early in development during symmetric divisions of neuroepithelial stem cells, however its loss specifically in RGCs was also shown to give rise to a thinner cortex with a less-organized structure. In the same study Lis1 was found to be important for localization of its binding partners Nde1-like (Nudel or NDEL1), dynein, and CLIP-170, and this localization was important for MT stability and capture at the cell cortex (Yingling et al., 2008; Moon et al., 2014). Related to this, protein phosphatase PP4c is required for proper asymmetric cell division in *Drosophila* neuroblasts, and conditional knockout mice were found to have thinner and disorganized cortical layers again partly related to spindle orientations that favor progenitor exhaustion. Indeed, PP4c can dephosphorylate Ndel1 and regulate its interaction with Lis1. Excessive phosphorylation of Ndel1 upon PP4c loss leads to disruption of the Ndel1/Lis1 complex and subsequent spindle orientation defects (Xie et al., 2013). As mentioned above, dynein, together with Kif1a and Lis1, has also been studied in progenitors in relation to mechanisms governing nuclear translocation during INM (Tsai et al., 2005, 2010). Recently mutations in *DYNC1H1*, encoding dynein heavy chain, together with *TUBG1*, *KIF5C* and *KIF2A*, have been associated with complex cortical malformations and microcephaly (Poirier et al., 2013), however, the mechanisms giving rise to these cortical malformations are still unclear. Even if they have not been found mutated in cortical malformation patients, other genes (e.g., AGS3, Vangl2, Table 2) have been described to play a role in mitotic regulation of RGCs and have been studied in mouse models with similar phenotypes (Montcouquiol et al., 2003; Sanada and Tsai, 2005; Lake and Sokol, 2009). These combined data underline a critical role of MTs and associated proteins during RGC mitosis, maintenance of RGC morphology, as well as during neuronal migration.

Another protein found to regulate spindle orientation, this time favoring basal progenitor production, is *Inscuteable* (Kraut et al., 1996). The mouse homolog, mInsc, is enriched at the spindle midzone in anaphase cells. Gain and loss of function experiments showed that when mInsc was knocked down the cortex was thinner, whilst mInsc overexpression led to a thicker cortex (Postiglione et al., 2011). A similar example is the Wnt3a overexpression mouse model (Munji et al., 2011). Loss of function mutations of Wnt3a lead to dramatic disruption of cortical development (Lee et al., 2000). Overexpression of Wnt3a in RGCs caused the formation of ectopic neuronal rosettes adjacent to the ventricle, and a dramatic increase in Tbr2-positive cells present in disorganized clumps or organized in rosettes in an expanded SVZ adjacent to a heterotopic neuronal mass (Munji et al., 2011, see also Schmid et al., 2014 for a similar phenotype). There was also RGC hyper-proliferation and unusual rosette organization, increasing the thickness of the VZ, similar to that observed in Lgl1 mutants (Klezovitch et al., 2004; Table 2), making a further link hence to polarity and adhesion complexes. Cdc42 deletion in RGCs also caused an increase in the number of basal progenitors (Table 2), but in this case the consequence was an increased neurogenesis and the formation of a thicker cerebral cortex, without the formation of rosettes or heterotopia (Cappello et al., 2006). Indeed, mechanisms determining the different progenitor outcomes in these cases are not yet elucidated.

Most genes mentioned so far in this section make the relationship between defects in spindle function and a depletion of RGC progenitors, associated with either premature differentiation, increased basal progenitors, or increased cell death. However, there are also models where spindle mis-orientation is found concomitant with misplaced progenitors that remain Pax6-positive and potentially maintain the ability to produce all cell types in an ectopic position. Several mutants with detached Pax6 progenitors, in which spindle orientation was not necessarily previously studied, were also mentioned in the previous sections. If detached Pax6-positive cells survive, this mechanism can either lead to a thinner, or a thicker cortex, or to subcortical heterotopia where ectopic masses of cells remain present in the white matter, but the overall apical-basal architecture of the cortex seems to be maintained. We discuss here certain mutants which can help us begin to understand such phenotypes. In Pax6 mutants, altered spindle orientation and cleavage planes in RGCs resulted in a markedly unequal inheritance of the ZO1-labeled adherens junction components and the apical membrane domain enclosed by these. Non-apical cell divisions were found increased in the mutant cortex, and most of the basally dividing cells retained RGC hallmarks consistent with premature delamination (Asami et al., 2011). PAX6 mutations in patients lead to aniridia and complex malformations (OMIM 607108). Also, *LGN* (G-protein-signaling modulator 2, GPSM2) codes for a G-protein regulator that links the cell cortex and mitotic spindles (Du et al., 2001; Du and Macara, 2004). LGN protein was found concentrated on the apical side of the VZ and localized to the lateral cell cortex in dividing apical progenitors (Konno et al., 2008). In *LGN*-mutants, mitotic orientations of progenitors were essentially randomized at E10.5 and E14.5, and Pax-6- and Tbr-2-positive cells were scattered into the SVZ and IZ. Non-surface apical progenitors were formed at the expense of attached RGCs, causing a decrease of approximately 30% in the thickness of the VZ. The average length of the progenitors’ cell cycle and the production of neurons were unchanged in the mutant (Konno et al., 2008). Thus, changes in spindle orientation can cause detachment and misplacement of progenitors without apparently changing their identity. Although this model does not give clues about an overall resulting malformation, another similar model displaying ectopic Pax6-positive progenitors scattered in the VZ and IZ, is represented by the *HeCo* (*Heterotopic Cortex*) mouse (Croquelois et al., 2009). The gene mutated in this model is *Eml1*, coding for an MT-associated protein whose function in brain development is not known. *EML1* was found mutated in patients affected by a very severe form of periventricular and globular ribbon-like subcortical heterotopia, and *HeCo* mice show a similar phenotype to band heterotopia (Kielar et al., 2014). The spontaneously arisen *tish* rat model (Lee et al., 1997), and BXD29 mouse mutants (Croquelois et al., 2009; Rosen et al., 2013), with unknown mutations, also show a similar subcortical heterotopia. Certain other mutants mentioned above, e.g., conditional knockout of RhoA (Cappello et al., 2012) or overexpression of mInsc or Wnt3a, also have subcortical neurons, although they show either a severe displacement of the VZ and defects in the ventricular lining, or greatly increased numbers of basal progenitors, and hence causative mechanisms may not to be identical. In the case of the *HeCo* mouse, like LGN mutants, the VZ is largely intact although slightly reduced in thickness and only a proportion of Pax6-positive progenitors show a re-distribution into the SVZ and IZ (Kielar et al., 2014). Tbr2-positive basal progenitors are also found ectopically, although do not differ in overall number in the *HeCo* model. Human patients with mutations in EML1 also exhibit macrocephaly, in some cases associated with hydrocephaly. The analysis of the *HeCo* model showed defects in mitotic spindle orientation during the neurogenic period that might be the cause of the detachment of some apical progenitors from the VZ, which is likely to be the primary event which eventually leads to heterotopia formation in this case (Kielar et al., 2014). Perturbed RGC guides almost certainly contribute to the phenotype. It is hence clear from these and other data that subcortical heterotopia can arise via multiple mechanisms.

#### Cytokinesis

Another important step during progenitor division is cytokinesis, the final separation of the two daughter cells. RGCs divide using a polarized form of cytokinesis, which is not well understood. Cytokinetic furrowing starts on the basal side and ingresses toward the apical membrane, where the midbody is formed. Cytokinetic abscission is mediated by the midbody at the ventricle, only after the daughter nuclei have migrated away (Kosodo et al., 2008). The midbody is a structure that forms at the end of the furrow and it contains central spindle compacted MTs and other factors important for mediating abscission. Moreover, midbodies of progenitors have been shown, together with the primary cilium, to release extracellular membrane particles enriched in the stem cell marker Prominin-1, thus influencing the balance between proliferation and differentiation after division (Dubreuil et al., 2007).

Spindle functioning and cytokinesis are tightly linked processes, this explains why proteins which function at the spindle poles or at the spindle midzone are often found also at the midbody (e.g., dynein, Horgan et al., 2011). Although this has not been widely studied, another protein that seems to function at both spindle poles and the cytokinetic furrow is Aspm, involved in microcephaly. As mentioned above, Aspm has been shown to be involved in regulating the precise cleavage plane orientation required for symmetric, proliferative divisions (Fish et al., 2006). This protein was also found enriched at the midbody of neuronal progenitors and thus, it was hypothesized to coordinate spindle rotation with cell abscission. Another example is the *magoo* mouse mutant, carrying a recessive, perinatal lethal mutation in the *Kif20b* gene, with fully penetrant microcephaly (Janisch et al., 2013). The thickness of the cortex is reduced in these mutants, but the layered structure is preserved. The output of progeny by apical progenitors is greatly reduced, but their capacity to produce daughters with ordered layer fates is intact. Kif20b protein was detected in cytokinetic midbodies of progenitors at the ventricular surface, where it is thought to regulate midbody behavior by transporting different cargoes. In the *magoo* mutant, midbodies had an abnormal shape and appeared misaligned with respect to the ventricular surface, indicating defects in midbody formation, maturation or maintenance (Janisch et al., 2013). Thus, failures in abscission of progenitors are likely to have similar consequences as failures in mitotic spindle functioning, leading to microcephaly.

## Discussion

We discussed in this review how different types of cortical malformation arise following perturbations in RGC structure and/or mechanisms (mouse mutant data schematically resumed in Figure 2). Due to the large number of players involved (Figure 3), the resulting phenotypes are sometimes difficult to classify into distinct categories and a certain degree of overlap remains. Indeed, mutations in a single gene can be causative of several distinct malformations, and conversely, single malformations can be linked to mutations falling in genes apparently involved in different pathways or mechanisms. Here we classified genes in areas where they have been shown to play a major role, making a link where possible, with resulting malformations. However, minimal modifications regarding one compartment are likely to have dramatic secondary effects. It is hence possible that some mutants may not yet have been explored enough in order to identify primary vs. secondary events.

### Basal process

We discussed how perturbations of the RGC basal process mainly lead to heterotopia phenotypes, polymicrogyria and type II lissencephaly. These perturbations can be caused by mutations that affect BM components and the attachment of glial end-feet to the pial surface, e.g., glycosyltransferases and laminins. The phenotypes can be related to a cause or a consequence of basal process detachment and disorganization, for example, neurons may not find an appropriate scaffold for migration and are prone to generate heterotopias. Also, similar phenotypes are revealed in the mouse due to mutations of proteins that have a more ubiquitous role in RGC structure suggesting that some ECM interactions in apical and basal regions are similar. Mutations in genes like *GPR56*, genes encoding integrin subunits or components of the integrin signaling system, genes like *RhoA*, and *Marcks*, not only disrupt the basal process but have a wider, and perhaps primary, effect influencing apical attachment and progenitor proliferation. Although we classified certain proteins as perturbing the basal process, since the malformations generated are consistent with this, it makes sense in the future to systematically examine both extremities, and at early time-points. Similarly, the mechanisms leading to polymicrogyria are still under debate, but both the basal and apical attachments, and proliferation, are probably involved. Experiments aiming to film the detachment of RGC processes *ex vivo* may help to clarify the temporality and causality of apical and basal process defects.

### Apical domain

The apical domains of RGCs are even more complicated to dissect in terms of mechanisms leading to malformations. This domain is not only important for progenitor attachment to the ventricular surface, mechano-transduction, and maintenance of polarity, but it is also the place where primary cilium and junctional complexes between cells are located. Several proteins play a role in intercellular and ECM contacts in the VZ, and perturbations of their function mainly lead to ventricular lining breaks and PH. Sometimes these seem to be produced only by disorganization of RGCs, without altering the cell cycle and cell fate, such as for example after β1-integrin, laminin-α2, and FLNA mutations. More frequently, alteration of the junctional complexes involve proteins that are linked with signaling pathways and this not only produces disorganization but also affects cell cycle characteristics and the balance between proliferation and differentiation. Examples are mutations in Dchs1, Fat4, Mtll4 and Ephrin-B1. The primary cilium is a structure important for both mechano-transduction and signaling, and perturbations of this organelle lead to a special category of diseases called ciliopathies, which can include severe cortical disorganization, as mentioned in the case of Arl13b.

Disruption of proteins and complexes that form the apical polarity domain of RGCs have not been found in human cases of cortical malformations but studies in mice have clearly shown that they are important for the maintenance of ventricular lining integrity and the regulation of cell fate. Indeed, the αE- and β-catenin, Numb, Numbl, Par-complex components, their regulators such as ASPP2, and proteins such as Lgl1, are important to regulate the balance between progenitor expansion and neuron production (see Table 2). Moreover, the integrity of the VZ is often severely perturbed due to the close link these proteins have with adherens junctions and a potential role in the maintenance of their integrity. Cell biology studies could be further performed to dissect the exact role polarity proteins exert in the maintenance of adherens junctions between RGCs, this could help clarify why their mutations have consequences on cell fate. Indeed, a study in *Drosophila* neuroepithelium showed that Par-complex proteins act by regulating the endocytosis of molecules important for adherens junction stability (Harris and Tepass, 2008). This is an interesting area to pursue in the future.

### Mitosis, spindle function and cytokinesis

Due to the complexity of the mitotic machinery and the mitotic cycle, there are a number of steps potentially susceptible to perturbation and the exact mechanisms leading to cortical malformations are still difficult to dissect. Centrosomes are key structures, and mutations in associated proteins cause microcephaly in human as well as in mouse models. However, even if all the studied proteins have a role at the centrosome, the causes of the reduced production of neurons are not always the same. Indeed, in some cases, such as for example when proteins like MCPH1, STIL, and ASPM are mutated, a thinner cerebral cortex seems to be produced as the consequence of a premature exhaustion of the progenitor pool and premature neuronal differentiation. In these cases, premature differentiation may be the consequence of centrosomal perturbations that lead to spindle orientations that favor asymmetric divisions and cell detachment from the ventricular lining. In other cases, such as for example when CDK5RAP2, CENPJ, and PLK4 are mutated, microcephaly seems to be the product of an increased cell death affecting both the progenitor and neuronal populations, due to mitotic delay and/or aneuploidy. In these cases spindle mis-orientation may be a non-specific feature. The same malformation can hence arise due to potentially different mechanisms. Moreover, the different proteins can have an important role during different temporal windows and compensation mechanisms can also influence the different outcomes. A common feature of the two mechanisms is the elongation of M-phase, hence further studying cell cycle regulation may clarify why in some cases cell death is triggered and in other cases differentiation is favored.

We discussed proteins that regulate mitosis in RGCs without being specifically restricted to centrosome function. These proteins frequently show a localization that changes during the mitotic cycle. They can be involved in the regulation of the mitotic spindle at the poles influencing astral MTs and their interaction with the cell cortex, in the regulation of spindle MT assembly and dynamics, in chromosome congression at the metaphase plate, or in the generation of pulling and pushing forces necessary for chromosome segregation. What appears clear is that spindle orientation defects are always present when the mitotic process is affected, thus it appears as a consequence common to different underlying mechanisms, and probably not something specific to a certain malformation. The degree of the spindle orientation defect is, however, another variable that can influence the downstream cascade of events leading to a particular malformation. Cortical disruptions that derive from mutations in genes such as *NDE1*, *LIS1*, *PP4c*, *WDR62*, *CENPE*, *TCOF1*, *AGS3*, *VANGL2* and *HTT*, are classified as micro- or macro-cephaly, and/or cortical disorganization and abnormal cortical architecture, lissencephaly and simplified gyral patterns. These are in general due to progenitor detachment, mis-regulation of cell death and changes in cell fate, sometimes associated with abnormal neuronal migration. In the case of models like mInsc and Wnt3a overexpression, the common feature is an increase in indirect neurogenesis through the generation of basal progenitors, leading to heterotopia and an increase in cortical thickness. The reason why sometimes there is only a defect in the number of neurons produced without alterations of cortical laminar architecture, and sometimes the defects in mitosis lead also to an incapacity of the neurons produced to fit in a layered structure, is still not clear. One of the causes of additional problems in lamination are likely to be the presence of anomalies of the RGC scaffold, or due to multiple roles of these proteins during corticogenesis. Moreover, we can imagine that different variables influence these phenotypes: different proteins have distinct roles and severities once perturbed, also temporal issues can play a role, together with compensation mechanisms that do or do not take place. Moreover, we must consider that different types of cell integrate in a highly complex general architecture, and their mutual interaction is a very influential variable. Indeed, basal progenitors and neurons, can communicate with RGCs to regulate their mode of division through signaling pathways, such as for example Notch signaling (Nelson et al., 2013) that also influences transcription factors and genetic regulation, having also a consequence on neuronal identity, essential for lamination. Also, regulatory inputs from different brain areas (e.g., the thalamus (Gerstmann et al., 2015)) during development may influence progenitor behavior at specific time-points. However, this area remains for the moment little explored.

We also presented genes such as *PAX6*, *LGN* and *EML1* that have been found, when mutated, to lead to progenitor detachment from the ventricular surface that nevertheless appears intact. Ectopic Pax6-positive progenitors hence constitute a source of cells that can be the cause of heterotopia formation. Interestingly, in these mutants, spindle orientation defects and progenitor detachment do not lead to premature differentiation as in the case of microcephaly genes. Indeed progenitors that leave their apical position retain their proliferative capacities and RGC identity. What we can hypothesize is that for reasons that still remain unclear, detachment of a proportion of RGCs from the apical surface occurs in a way that allows them to retain the molecular signatures necessary to not exit the cell cycle and remain in a similar proliferative state. These mechanisms deserve further studies to try to understand why, in some cases, loss of apical attachment is accompanied by a switch to a state more committed to differentiation, and in some others this does not happen. The explanation can be sought also in the steps that regulate cell cycle progression and in the signaling with other cell types. For example, detached progenitors could be insensitive to signals that tell a cell outside the VZ to become a neuron. Conversely, the environment could change and with it the signal. Also, in these models detached progenitors could be insensitive to cell death pathways activated in some microcephaly models, when centrosomes are absent or are too numerous. Indeed, in the CENPJ and PLK4 studies it has been shown that once the tumor suppressor p53 is inactivated, ectopic progenitors actively divide in the IZ, suggesting a role for the centrosome in the anchoring of progenitors, but not in their ability to divide once detached.

Cytokinesis is the last step of cell division, and its perturbation was also shown to produce defects similar to spindle malfunction. The midbody is a transient structure that forms during cytokinesis and can contain molecular signatures important for cell fate determination of daughter cells, which is probably why changes in its structure or function potentially produce cortical malformations. Cytokinesis and its molecular players in RGCs still remain poorly studied mechanisms that deserve further investigation. Molecules that take part in mitotic regulation are often later found located at the midbody, however their accumulation for degradation, or alternatively importance in this structure are still little understood.

## Conclusions

In this review we hence correlated studies in mouse models and genes found mutated in cortical malformation patients. A large number of mutated proteins found to perturb cortical development in mouse models have not been identified in human. This could be sometimes due to the lethality of mutations in certain proteins in human, or simply to the fact that they remain to be identified by patient screening. In other cases, genes found mutated in patients have been studied in mouse models where they reproduce with different degrees the defects found in human. In rare cases mutations appeared spontaneously (e.g., *HeCo*) and were able to recapitulate, even if with certain differences, the phenotype found in patients carrying mutations in the same gene. In most cases, mouse models are generated by knocking-down genes found mutated in human diseases. This approach is being systematically generalized (e.g., international mouse knockout and phenotyping consortiums) as it furnishes an essential and very useful tool to be able to dissect the basic mechanisms leading to malformations studying the role of single proteins and integrating them in more complex networks. For this latter approach, mouse models furnish a good tool to perform genetic studies at a bigger scale through genome wide comparative studies in order to identify protein and/or regulatory networks important for normal and pathological brain development. These data will be further complemented in the future by knockin studies whereby individual mutations (e.g., missense) are studied in the rodent, to more closely match human pathological situations, and these studies will be extremely interesting to compare with previous knockout data.

Even if mouse models remain a very good tool to study human cortical malformations, such studies still remain a matter of debate as very often the consequences of mutations in the same gene are different when comparing the two organisms. With respect to this, it is important to consider essential differences between the mouse and human brain that can account for the different severities certain malformations have in the two organisms. The human cerebral cortex is much more complex and presents not only a much more extensive tangential and radial expansion but is also characterized by the presence of gyri and sulci. A larger number of progenitors is essential to produce this expansion and the interplay between the different cell types is different and more complex. Even if the classification of the different progenitor types in gyrencephalic species probably deserves further exploration, we know that the human cortex presents additional germinal layers, such as the inner and outer SVZ (i/oSVZ) that are located basal to the VZ (Fish et al., 2008; Borrell and Reillo, 2012). So far we know that in the oSVZ the so called outer or basal RG-like cells (oRGs or bRGs) are located and contribute to neuron production (Hansen et al., 2010). These cells are Pax6-positive but lack an apical attachment to the ventricular surface and have recently also been identified in small numbers in the mouse brain, however their role is still under debate (Shitamukai et al., 2011; Pilz et al., 2013). Such cells have rarely yet been studied in mouse models, but recently, the ventral telencephalon, the region of the murine brain with the largest SVZ, has been shown to be a useful model to study progenitor expansion. Indeed, novel and morphologically heterogeneous progenitor types have been identified in this area that can be traced to gyrencephalic cortices (Pilz et al., 2013). The process of gyrification also adds a degree of complexity to human cortical development and only recently some molecules have been identified potentially playing a role in this process (e.g., Trnp1, Stahl et al., 2013). Mouse models were shown to be useful in this respect, together with gyrencephalic species (e.g., the ferret). Thus, lissencephaly, as a characteristic of the mouse brain, can be viewed as a tool to study single proteins or networks that contribute to the formation of gyri and sulci in more evolved species. Recently, a three-dimensional culture system generating cerebral organoids has also been shown to represent a sensational tool to model human brain development and related cortical malformations (Lancaster et al., 2013). Similar studies will almost certainly help to fill the gap between mouse and human, and will contribute to the study of cortical malformations in the future.

## Author contributions

Sara Bizzotto and Fiona Francis wrote the review.

## Conflict of interest statement

The Review Editor Veronique Marthiens declares that, despite being affiliated to the same institution as authors Sara Bizzotto and Fiona Francis, the review process was handled objectively and no conflict of interest exists. The authors declare that the research was conducted in the absence of any commercial or financial relationships that could be construed as a potential conflict of interest.
